# Carbonylation accumulation of the *Hypsibius exemplaris* anhydrobiote reveals age-associated marks

**DOI:** 10.1371/journal.pone.0208617

**Published:** 2018-12-26

**Authors:** Mira Kuzmic, Myriam Richaud, Pierre Cuq, Sandrine Frelon, Simon Galas

**Affiliations:** 1 IRSN/PSE-ENV/SRTE - Laboratoire d’écotoxicologie des radionucléides, St Paul lez Durance Cedex, France; 2 IBMM, University of Montpellier, CNRS, ENSCM, Montpellier, France; University of Massachusetts Medical School, UNITED STATES

## Abstract

Together with nematodes and rotifers, tardigrade belong to micrometazoans that can cope with environmental extremes such as UV and solar radiations, dehydration, supercooling or overheating. Tardigrade can resist the harshest conditions by turning to cryptobiosis, an anhydrobiotic state that results from almost complete dehydration and is characterized by an ametabolic status. Although reports have challenged the molecular basis of the mechanisms underlying genomic injury resistance, little is yet known regarding the possible involvement of other tardigrade macromolecules in injury during a stress experience. In this report, we show that the tardigrade *Hypsibius exemplaris* can accumulate molecular damages by means of *in situ* detection of carbonyls. Furthermore, we demonstrate that living tardigrade can accumulate carbonylation. Finally, we reveal that anhydrobiotic tardigrade can be constitutively affected by carbonylation that marks aging in other metazoans.

## Introduction

Tardigrade are minute metazoan animals, ranging from 0.1 to 1.2 mm in size, that inhabit diverse habitats such as freshwater, marine or the water films of terrestrial moss and lichens. Approximately 1000 species of tardigrade have been identified to date [1–3], and several have been reported to have the ability to cope with the harshest environments [4]. For example, they can survive up to 10 kJ/m2 UV exposure [5], a ten-day space flight exposure to solar radiations at low earth orbit in the space vacuum [6], or exposure of up to 7.5 GPa pressure [7], which equals the pressure at a depth of up to 180 km from the earth’s surface. Moreover, tardigrade can withstand treatment with organic solvents [8], extreme temperatures (ranging from -272 to 151°C) or high radiation doses (kGy) [9].

However, all these observations of tardigrade extreme resistance have been made during a latent stage of life [10–12] that forms by dehydration and allows for the resistance to desiccation. When they lose their surrounding water film, terrestrial tardigrade initiate contractions along their anterior-posterior body axis and retract their limbs to form an anhydrobiotic organism with a characteristic “tun” shape. Tardigrade can then lose up to 97% of their bound and free body water content during the anhydrobiosis process [13–15]. Together with small organisms, such as rotifera and nematoda, tardigrade can enter anhydrobiosis at any stage of their life cycle [16–22].

Reactive oxygen species (ROS) originate from intracellular sources, mainly mitochondria, and are biproducts of energy metabolism. ROS are highly unstable oxygen derivative molecules that can damage, by oxidation, various intra-cellular molecules such as lipids, proteins, sugars and nucleic acids (DNA, RNA) either directly or indirectly, thereby reducing the normal activity and function of numerous macromolecular components [23]. Thymidine dimer formation is believed to be the main DNA injury caused by UVC [24]. Moreover, gamma radiation and UV light can induce ROS formation [25,26], resulting in damaged cellular macromolecules. For example, up to 70% of the DNA damage can be attributed to ROS formation when water radiolysis occurs upon radiation [24]. The main ROS-target of tardigrade assessed to date concern the DNA molecule [27] but ROS-damaged proteins can also accumulate as carbonylated products which may impact normal biochemical activity and are associated with aging and human degenerative diseases [28,29].

Because of the admittedly high resistance of the tardigrade to several of the harshest treatments, we then assessed if the tardigrade *Hypsibius exemplaris* can accumulate carbonylations during the anhydrobiotic or fully active period. Here, we used a new technique that allows the detection of total carbonylated proteins [30]. Thereby, we showed that the tardigrade *Hypsibius exemplaris* can accumulate carbonylations during both the anhydrobiotic and hydrated periods. Furthermore, we uncovered a gradual accumulation of carbonylation as desiccated tardigrade aged.

## Results

### Carbonylation in active tardigrade induced by UVC

**Fig 1:**
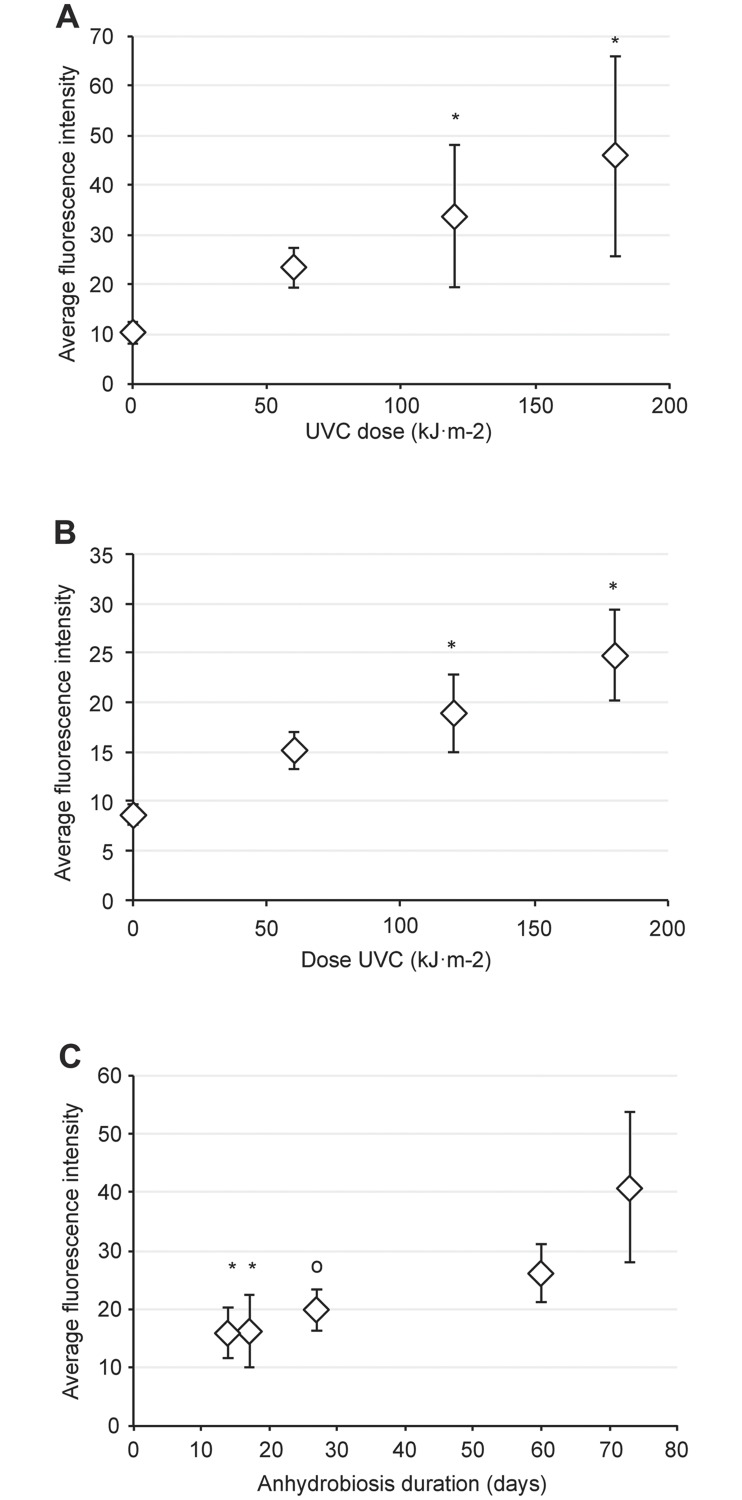
Carbonylation accumulation of the *Hypsibius exemplaris* tardigrade. Average intensity of the *in situ* carbonylation staining of active (A) and anhydrobiote (B) tardigrade in response to rising UVC doses or during anhydrobiote aging (C). Errors bars indicate the standard deviation from at least three repeated experiments. Total animals scored was n = 34 for (A), n = 20 for (B) and n = 37 for (C). The asterisk represents the respective significant difference of the Kruskall-Wallis test at *P*<0.05. The respective curve correlation degree are y = 0.195 x + 10.75; R^2^ = 0.998 for (A), y = 0.087 x + 9.07; R^2^ = 0.990 for (B) and y = 0.36 x + 9.92; R^2^ = 0.88 for (C). The respective Spearman’s correlation coefficient are *P*<0.05, *ρ* = 0.76 for (A) and *P*<0.05, *ρ* = 0.91 for (B). The mini-circle marks a less significant difference (Kruskall-Wallis’s test, *P*<0.1) with carbonyl accumulation of the desiccated tardigrade at 73 days (C).

Living tardigrade exposed to rising doses of UVC revealed a carbonyl content increase (Fig 1A). We measured a high correlation degree (linear regression analysis: y = 0.195 x + 10.75; R^2^ = 0.998; *P*<0.05 and Spearman correlation coefficient *ρ* = 0.76) between the carbonylation detection and the UVC dose of the irradiated group. Moreover, while the carbonyl accumulation with a 60 kJ.m^-2^ UVC irradiation dose did not differ significantly from the control, both UVC dose 120 kJ.m^-2^ and 180kJ.m^-2^ showed a significant difference (Kruskall-Wallis test, *p*<0.05). With the fluorescence background staining of the control group, we observed an almost 5-fold increase in the carbonylation staining for the 180 kJ.m^-2^ irradiation condition (Fig 1A). Interestingly, the carbonyl content detected did not plateau between the 120 kJ.m^-2^ and 180 kJ.m^-2^ irradiation doses (Fig 1A). This observation indicates that perhaps the accumulation of UVC-related carbonylation damage may override the highest measured level in the present study.

**Fig 2:**
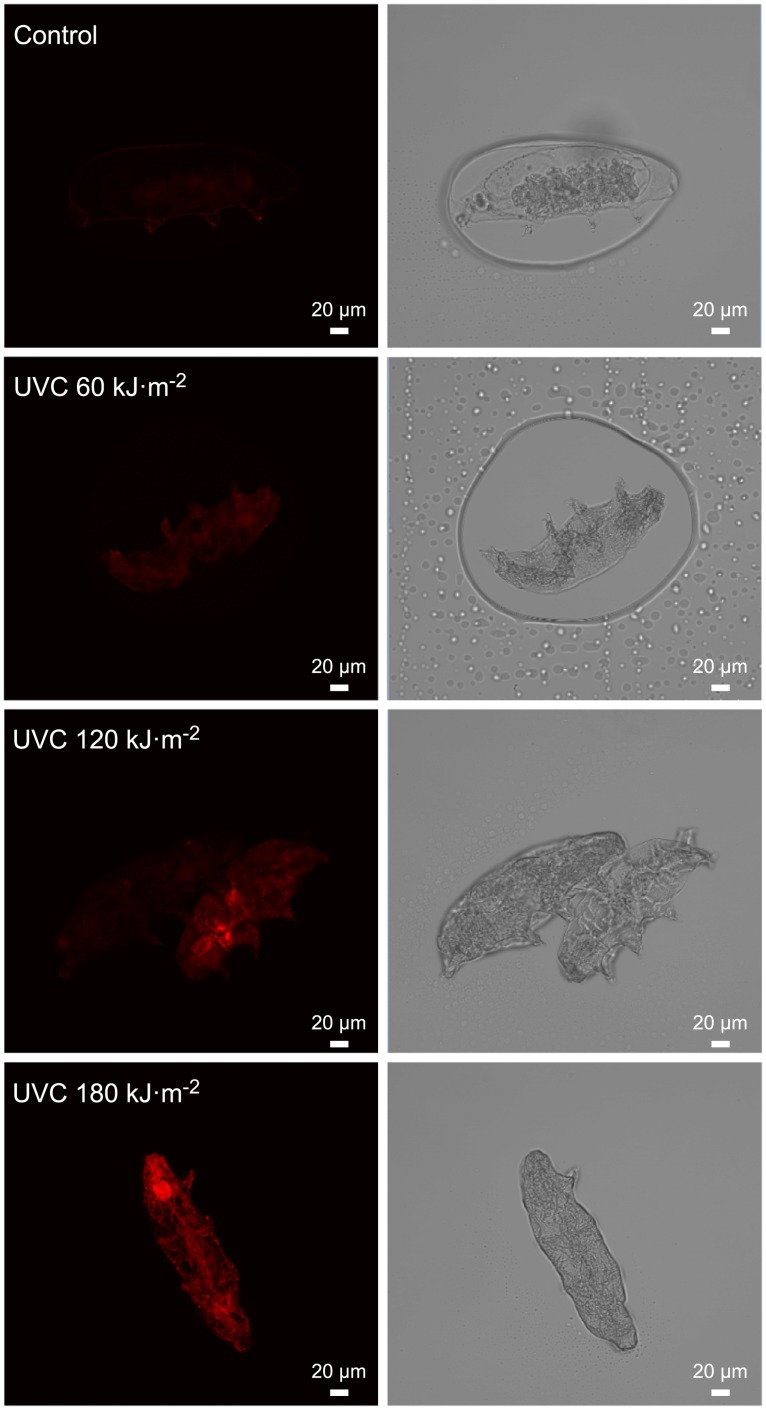
Confocal detection of carbonylation upon exposure of the tardigrade to rising UVC doses during the hydrated state. The pictures are representative of the three independent experiments depicted in Fig 1A. Scale bar = 100 μm.

Fig 2 shows representative images of direct *in situ* carbonyl labeling. We detected a faint carbonylation signal when active tardigrade were irradiated with 60 kJ.m^-2^ UVC (Fig 2B). However, *in situ* carbonylation staining progressively strengthened with 120 (Fig 2C) and 180 kJ.m-2 (Fig 2D) UVC irradiation doses, illustrating the correlation between the delivered UVC irradiation dose and carbonyl staining. Moreover, no active tardigrade survived exposure to the UVC radiation doses assessed here.

### UVC irradiation can induce carbonylation in anhydrobiotes

Anhydrobiotic tardigrade result from physiological and molecular modifications at a deep level. We then assessed if dehydrated tardigrade can accumulate carbonyl signatures to the same extent as the active tardigrade. We observed a positive link between the rising dose of UVC exposure and the carbonyl content in desiccated tardigrade (Fig 1B). Furthermore, we noticed a high degree of correlation between the UVC radiation dose and the carbonyl signal (linear regression analysis: y = 0.087 x + 9.07; R^2^ = 0.990 and Spearman correlation coefficient *P*<0.05, *ρ* = 0.91). Moreover, similar to the living tardigrade groups observed in the above section, we detected a highly significant difference in the carbonyl content between the control group and the 120 kJ.m^-2^ or 180 kJ.m^-2^ UVC irradiated groups (Kruskall-Wallis test, *p*<0.05), which showed a 2- and 2.5-fold increase in carbonyl content, respectively. Conversely, the tardigrade group that received a 60 kJ.m^-2^ UVC radiation dose did not differ in carbonyl content from the control group.

**Fig 3:**
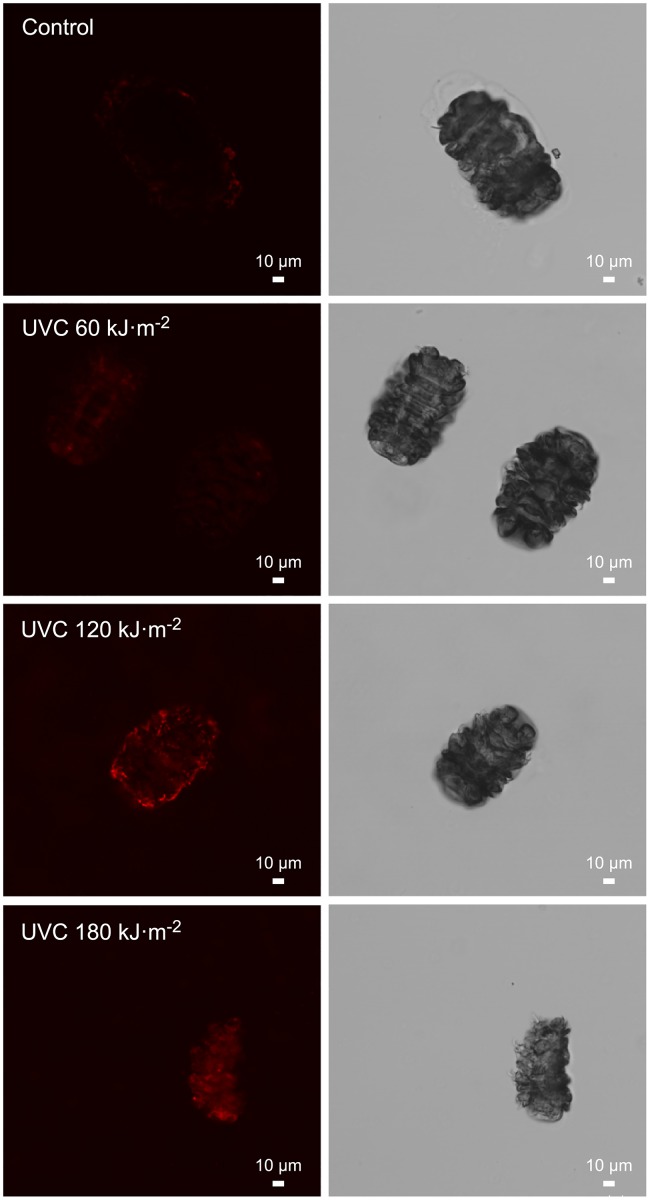
Confocal detection of carbonylation in the tardigrade anhydrobiotes in response to UVC. Representative images corresponding to the three independent experiments plotted in Fig 1B. Scale bar = 100 μm.

Fig 3 shows selected images of the direct *in situ* carbonyl staining of anhydrobiotic tardigrade. We observed a UVC dose-dependent increase in carbonyl labeling with a faint carbonylation staining for the 60 kJ.m^-2^ UVC irradiation dose (Fig 3B), followed by stronger carbonyl labeling for the 120 kJ.m^-2^ (Fig 3C) and 180 kJ.m^-2^ (Fig 3D) UVC irradiation doses.

Because of the absence of a plateau in the carbonyl staining curve observed between the 120 kJ.m^-2^ and 180 kJ.m^-2^ UVC irradiation doses, it is plausible that the maximal UVC irradiation dose assessed in this study did not allow for the highest possible carbonylation content of the anhydrobiotic tardigrade to be reached. Nevertheless, we observed up to a two-fold increase in total carbonylation (Fig 1B) in the irradiated groups of desiccated tardigrade compared to that in the control group of desiccated tardigrade.

### UVC impacted both the survival and progeny hatching of anhydrobiotic tardigrade

**Fig 4:**
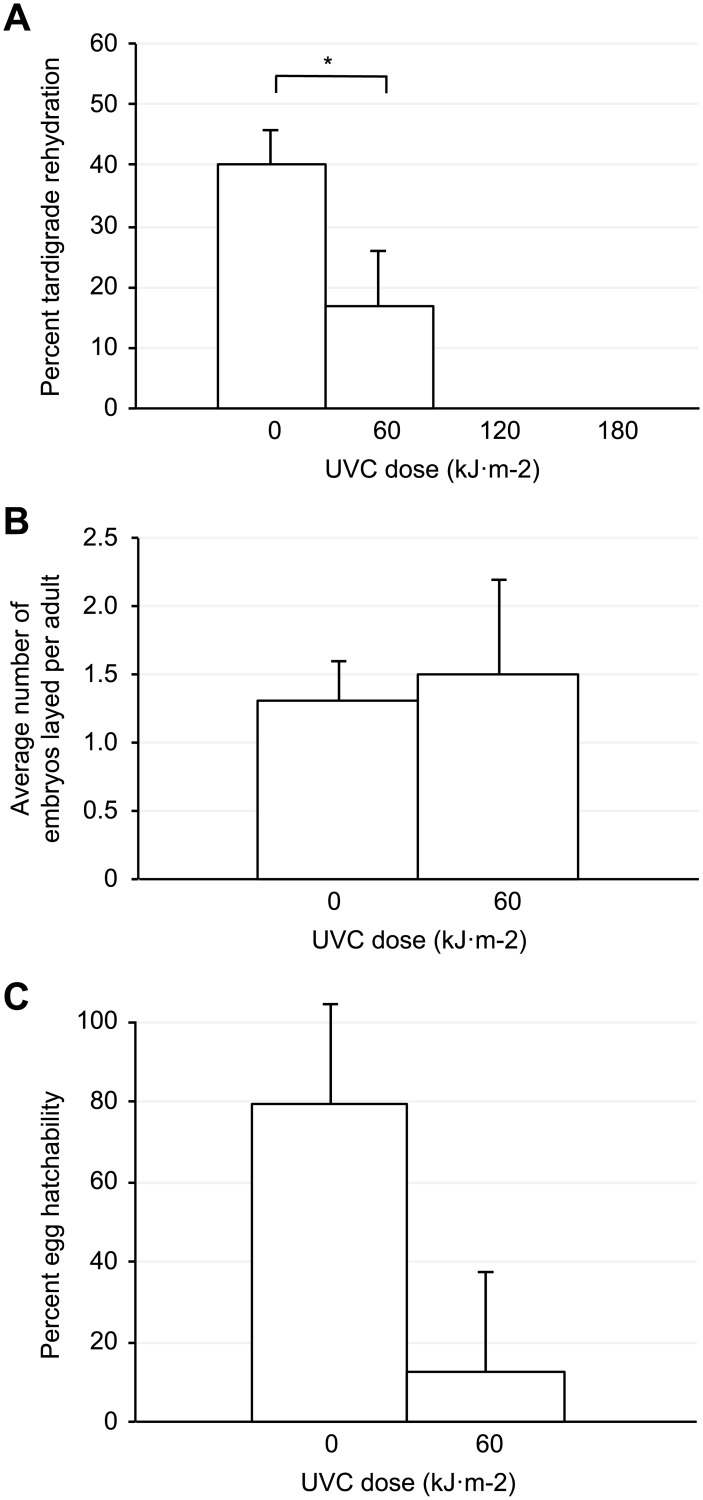
Survival and progeny hatching of *Hypsibius exemplaris* tardigrade irradiated with UVC during anhydrobiosis. (A) Mean percentage of survival after UVC irradiation and rehydration. Error bars represent standard deviation (n = 200). The asterisk indicates respective significant difference with control (Pairwise Z-test, *P*<0.05, *p*-value = 0.029). (B) Average number of embryos laid per rehydrated tardigrade. The error bars represent standard deviation (n = 37). (C) Mean percentage of the embryos hatchability. Error bars represent standard deviation (n = 37).

The UVC-dependent carbonyl measured in the anhydrobiotes prompted us to evaluate their possible impact during the tardigrade rehydration process. Fig 4A shows the anhydrobiotic exit of desiccated tardigrade subjected to various UVC irradiation doses. We observed that compared with the rehydrated and active tardigrade of the control group, the rehydrated and active tardigrade of the 60 kJ.m^-2^ UVC dose group diminished by a two-fold factor. Moreover, a comparison between both tardigrade groups showed a significant difference (Paired Z-test, *P*<0.05, *p*-value = 0.029).

Likewise, we noticed that the time needed for the tardigrade group subjected to the 60 kJ.m^-2^ UVC irradiation dose to fully rehydrate was postponed by 19 hours compared with that needed for the control group. This means that perhaps UVC irradiation either directly or indirectly impaired the anhydrobiotic exit process.

In contrast, both 120 and 180 kJ.m^-2^ UVC irradiation doses resulted in a total failure of the anhydrobiotes to fully rehydrate, and these groups died. However, we recovered a unique individual from the group of dehydrated tardigrade irradiated with the 120 kJ.m^-2^ UVC dose who started rehydration with a normal appearance but instead retained an uncommon gray color and a failure to regain any motility until 5 days post rehydration.

As we have shown, the anhydrobiotic tardigrade group irradiated with the 60 kJ.m^-2^ UVC dose yielded a survivor fraction during the rehydration process assay (Fig 4A). Based on this observation, we then assessed if the brood size was also affected. As shown in Fig 4B, we observed comparable egg production in both the control and the 60 kJ.m^-2^ UVC irradiated tardigrade groups.

However, even though it was normal in appearance, egg production can be concomitant with an impairment in the egg hatchability. Fig 4C depicts the hatchability of the eggs laid by the control and 60 kJ.m^-2^ UVC irradiated groups. We detected a drastic decrease in the hatchability of the eggs laid by the irradiated tardigrade group compared with those laid by the control group (Fig 4C). However, we observed that the irradiated anhydrobiotes can produce viable embryos even after exposure to a high UVC dose up to 60 kJ.m^-2^.

### Constitutive carbonylation accumulation of desiccated tardigrade

In this report, we have shown that both hydrated and anhydrobiotic tardigrade can show a detectable carbonyl signature in response to the UVC irradiation doses that we assessed. We then investigated if the anhydrobiotic tardigrade may constitutively accumulate carbonyls. To conduct this investigation, we left groups of dehydrated tardigrade for different times (see Materials and methods section) and then subjected the groups to the *in situ* carbonyl detection labeling. As shown in Fig 1C, we observed that tardigrade anhydrobiotes progressively accumulated a carbonyl signature. Moreover, we determined that carbonyl labeling levels are well correlated with the time spent in the anhydrobiotic state. Thus, we noted that carbonyl labeling remained at approximately the same intensity level after both 14 (mean value 15.9) and 17 (mean value 16.3) days of anhydrobiosis (Fig 1C). Next, the carbonyl staining increased over 27 days (mean value 19.9) at 60 days (mean value 26.1) of anhydrobiosis, the staining reached almost twice the value observed at 14 days (mean value 15.9). Finally, at 73 days (mean value 40.8) of anhydrobiosis, we observed a value twice that of the one observed at 27 days (mean value 19.9) and almost three times the mean value observed at 14 days of anhydrobiosis (mean value 15.9).

We observed a significant difference (Kruskall-Wallis test *P*<0.05) between the carbonyl labeling intensity in both the 14 and 17 day anhydrobiotic groups in compared with that in the older 73 day anhydrobiotic tardigrade group. Furthermore and to a lesser extent, we observed a significant difference (Kruskall-Wallis test *P*<0.1) of the carbonyl labeling between the anhydrobiotic group aged 27 days and the older group aged 73 days. Altogether, the data plotted in Fig 1C showed the following linear regression analysis: y = 0.36 x + 9.92; R^2^ = 0.88.

In summary, we have uncovered an unexpected constitutive carbonyl accumulation in tardigrade anhydrobiotes.

## Discussion

Previous reports of total carbonyl analysis used methods that included sample extraction protocols from whole organisms [31]. However, these protocols have been challenged because of underestimation of carbonylation due to a failure to extract an aggregated protein fraction insolubilized by a UV-dependent mechanism [32]. Moreover, other reports used protocols with chemicals such as 2,4-Dinitrophenylhydrazine (DNPH) or paraformaldehyde (PFA), which are believed to induce a carbonylation overestimation [33,34].

To increase the sensitivity and the specificity of the carbonyl labeling, we adapted an improved *in situ* carbonyl staining technique [30]. This technical procedure can bypass any extraction protocol requirements and reduce spontaneously occurring carbonylation reactions prior to analysis. In a first set of experiments, we performed carbonyl labeling of hydrated tardigrade in response to rising UVC irradiation doses. We were then able to detect up to a five-fold increase in carbonyl staining in the irradiated groups with the maximal UVC dose assessed (180 kJ.m^-2^). Moreover, all the UVC irradiation doses that we assessed here were higher than the doses used by Horikawa et al. [35] and caused instant death of hydrated tardigrade.

By contrast to other tardigrade species that can withstand direct exposure to relatively low humidity environments, *Hypsibius exemplaris* needs preconditioning pre-exposure to a high humidity environment to fully develop the characteristics of a stress-resistant anhydrobiotic organism [36–39]. Nonetheless, to date, studies [40] have provided new insights into the stress resistance of *Hypsibius exemplaris* when hydrated but not as an anhydrobiote. Unexpectedly, we observed that anhydrobiotes of *Hypsibius exemplaris* could survive a high UVC dose (60 kJ.m^-2^). Moreover, we noticed that the egg production of the rehydrated animals, previously irradiated as anhydrobiotes with a 60 kJ.m^-2^ UVC dose, was not impaired.

In the next experiment, we assessed if the hatchability of the eggs produced by the anhydrobiotes irradiated with high UVC doses was affected. We observed that the hatchability of the eggs laid by the *Hypsibius exemplaris* groups irradiated with UVC (60 kJ.m^-2^) appeared drastically reduced (11%). This result is significantly different from the reported hatchability (80%) of *Ramazzottius varieornatus* eggs when irradiated with 20 kJ.m^-2^ of UVC [35]. This difference result perhaps from a higher sensitivity of the *Hypsibius exemplaris* embryo precursors to UVC radiation while inside the anhydrobiotes. Therefore, because the germ cell or embryo status during the *Hypsibius exemplaris* anhydrobiotic stage remains unknown, we cannot yet explain the possible impact of UVC on both fertility and hatchability. Previous studies on tardigrade irradiation were performed on isolated embryos [14,24,40,41–43] but we described here the effect of UVC on the hatching of embryos that were not directly irradiated but instead exposed while inside the anhydrobiotic tardigrade.

In the final experimental section, we were surprised to observe gradual, proportional increases in the carbonyl content of the *Hypsibius exemplaris* anhydrobiotes of different ages. Moreover, we were able to detect up to a 4-fold increase in carbonylation at 73 days of anhydrobiosis compared with that in controls. The degree of carbonylation observed is almost of the same order of magnitude as the maximum carbonylation level that we observed when living tardigrade were irradiated with 180 kJ.m^-2^ of UVC. Moreover, this increase of up to 4-fold of spontaneous carbonylation is well above the 2.5-fold carbonylation increase observed for anhydrobiotes exposed to the 180 kJ.m^-2^ UVC dose. This observation may indicate that tardigrade can accumulate higher carbonylation levels than those observed in this work.

Proteins are the main targets of oxidation by ROS, which can result in protein carbonylation accumulation. In this work, we showed that *Hypsibius exemplaris* can accumulate carbonylations. Based on the report describing the labeling technique also described here [30], it is likely that after UVC exposure, the carbonylation labeling observed is linked to proteins. A protein carbonylation increase has been reported with age in various species such as yeast [44], flies [45], worms [46] and mammals [47].

The carbonylation of proteins is a process recognized as the universal marker of oxidative stress that has been associated with several human disorders and diseases of aging [23]. Because an increase in protein carbonylation marks the *Hypsibius exemplaris* anhydrobiote as it ages, we propose that this species must experience aging. However, whether other tardigrade species also accumulate carbonylation as they age still needs to be assessed. The tardigrade *Hypsibius exemplaris* is not as resistant to harsh conditions as other tardigrade species. However, innovative molecular techniques [48] and recently available genome data [49–53] make *Hypsibius exemplaris* a good integrative model to decipher how tardigrade species can regulate carbonyl clearance and proteostasis.

## Materials and methods

### Tardigrade culture and desiccation/rehydration protocols

*Hypsibius exemplaris* tardigrade [54] were fed with unicellular algae *Chlorococcum* sp.; both were purchased from the Sciento company (Manchester, UK). Specimens were maintained in cultures at 15°C on Chalkley’s medium as previously described by Gabriel et al. [48].

The desiccation protocol for randomly selected adult tardigrade was adapted from Hengherr *et al*. [55] and conducted as follows: coverslips were placed at 25°C in a sealed preconditioning box with 85% relative humidity (RH) for 16 hours with a saturated solution of KCl. The coverslips were then placed into a sealed box at 33% relative humidity (RH) for 48 hours with an MgCl2 saturated solution. Full dehydration of the specimens was monitored by direct observation under a stereomicroscope. The rehydration of desiccated tardigrade on coverslips was initiated by a drop of Chalkley’s medium and the motility evidence of rehydrated animals was monitored by direct observation under stereomicroscope. The anhydrobiotes were stored at a relative humidity (RH) of 24% at room temperature until analysis. The brood size of the rehydrated specimens, either UVC irradiated or not, was monitored over a two-week period.

### UVC treatment and labeling of carbonyls in anhydrobiotic tardigrade

UVC radiation and carbonyl labeling were performed according to a previous protocol [30] but with an adaptation for tardigrade. UVC treatment of dehydrated tardigrade was performed *in situ* on slides for 10, 20 and 30 minutes with a dose rate of 99.99 J.m^−2^.s^−1^. To keep tardigrade dehydrated and fixed to their slide, we irradiated tardigrade *in situ* without adding any additional liquid, and the carbonyl labeling process, *i*.*e*., chemoporation, the labeling of carbonyls and washing, were performed in 100% (iPrOH). To keep the liquid on dehydrated tardigrade during the protocol and to enable the labeling of carbonyls, we cut a 1 ml pipette tip and placed it around tardigrade on slides. Chemoporation lasted for 3 minutes on ice (150 μL 100% isopropanol) and labeling of the carbonyls lasted 45 minutes with 4 μg.ml^−1^ Cy5 Hz (Cy5 Mono Hydrazide, GE Healthcare Life Sciences, Buckinghamshire, UK) in 100% iPrOH. Washing was performed in 4 cycles with 150 μl 100% iPrOH. Slides were prepared by pipetting a minimal volume of mounting medium (Eukitt Quick-hardening mounting medium, SIGMA-ALDRICH Co., St. Louis, Missouri, USA) to cover tardigrade and then placement of a cover slip.

### UVC treatment and labeling of carbonyls in living tardigrade

Radiation was performed in 1 ml M9 buffer (M9) in a 3 cm glass petri dish for 10, 20 and 30 minutes with a dose rate of 99.99 J.m^−2^.s^−1^. Tardigrade were transferred with a 10 μl pipette to a new empty tube in a minimal volume of buffer. Chemoporation was performed with 150 μl 100% iPrOH for 3 minutes on ice followed by tardigrade transfer. Labeling of carbonyls was performed with 150 μl 4 μg.ml^−1^ Cy5 Hz in 100% iPrOH for 45 minutes followed by tardigrade transfer. Washing was performed with 150 μl in 4 cycles with 40% iPrOH. Then, for each test condition, all tardigrade were pipetted with a P10 onto a slide, one drop of mounting medium was added, and then a coverslip was placed.

### Confocal microscopy imaging and fluorescence quantification

Images were obtained with an LSM 780 confocal microscope (Carl Zeiss, France) using a 10× dry (N.A. 0.45) and 20× dry (N.A. 0.80) or 63× oil (N.A. 1.4) oil immersion objective, with a pinhole setting of 1.31 A.U. and 8- or 12-bit images. Cy5 was excited with the red HeNe laser (633 nm), and emitted light was collected between 643 and 696 nm. Fluorescence quantification was also performed with a confocal microscope according to a previously described protocol [30].
